# Prevalence and risk factors for human papillomavirus infection among female sex workers in Hanoi and Ho Chi Minh City, Viet Nam: a cross-sectional study

**DOI:** 10.5365/wpsar.2022.13.4.894

**Published:** 2022-11-07

**Authors:** Quang Duy Pham, Kiesha Prem, Tuan Anh Le, Nguyen Van Trang, Mark Jit, Tuan Anh Nguyen, Van Cao, Tam-Duong Le-Ha, Mai Thi Ngoc Chu, Ly Thi Khanh Le, Zheng Quan Toh, Marc Brisson, Suzanne Garland, Gerald Murray, Kathryn Bright, Duc Anh Dang, Hau Phuc Tran, Edward Kim Mulholland

**Affiliations:** a Pasteur Institute of Ho Chi Minh City, Ho Chi Minh City, Viet Nam.; b Department of Infectious Disease Epidemiology, London School of Hygiene and Tropical Medicine, London, United Kingdom.; c National Institute of Hygiene and Epidemiology, Hanoi, Viet Nam.; d School of Public Health, University of Hong Kong, Hong Kong, SAR (China).; e Public Health England, Modelling and Economics Unit, London, United Kingdom.; f New Vaccines, Murdoch Children’s Research Institute, Parkville, Victoria, Australia.; g Department of Paediatrics, The University of Melbourne, Parkville, Victoria, Australia.; h Centre de recherche du CHU de Québec-Université Laval, Quebec, Canada.; i Department of Social and Preventive Medicine, Université Laval, Quebec, Canada.; j Department of Obstetrics and Gynaecology, The University of Melbourne, Parkville, Victoria, Australia.; k Centre for Women’s Infectious Diseases Research, The Royal Women’s Hospital, Parkville, Australia.; * These authors are joint first authors.; # These authors are joint last authors.

## Abstract

**Objective:**

Female sex workers (FSWs) are at high risk of human papillomavirus (HPV) infections and cervical cancer due to their high number of sexual partners. The objectives of this study were to determine the prevalence of HPV and identify risk factors for high-risk HPV infection among FSWs in Hanoi and Ho Chi Minh City (HCMC), Viet Nam.

**Methods:**

A cross-sectional study was conducted in Hanoi and HCMC between December 2017 and May 2018. We surveyed and screened 699 FSWs aged ^3^18 years for HPV infection and abnormal cytology. A multivariable modified Cox regression model was used to determine risk factors for high-risk HPV infection.

**Results:**

The overall prevalence of any HPV, high-risk HPV and HPV-16/18 infection in the 699 FSWs was 26.3%, 17.6% and 4.0%, respectively, and were similar in both cities. Multiple infections were identified in 127 participants (69.0%). HPV-52 was the most prevalent (7%), followed by HPV-58 (6%). Abnormal cytology was detected in 91 participants (13.0%). FSWs who are divorced (adjusted prevalence ratio [aPR]: 1.96, 95% confidence interval [CI]: 1.01–3.81), widowed (aPR: 3.26, 95% CI: 1.49–7.12) or living alone (aPR: 1.85, 95% CI: 1.01–3.39) were associated with a higher prevalence of high-risk HPV infection.

**Discussion:**

Almost one in five FSWs in Viet Nam are infected with high-risk HPV. This highlights the importance of prevention strategies such as HPV vaccination and screening in this high-risk group.

Cervical cancer, which is caused by persistent human papillomavirus (HPV) infection usually by oncogenic/high-risk HPV type(s), is the fourth leading cause of cancer mortality among women globally, with an estimated 570 000 new cases and 311 000 deaths in 2018. (*1*) The majority of these cases occur in low- and middle-income countries (LMICs), primarily due to the low uptake of HPV vaccination, lack of robust HPV screening programmes and limited treatment options. (*2*) In response to the global public health burden, in 2020, the World Health Organization (WHO) set a threshold of four cervical cancer cases per 100 000 women for the elimination of cervical cancer as a public health problem and launched the 90–70–90 targets, aiming to fully immunize 90% of girls against HPV by 15 years of age, screen 70% of women for cervical cancer by 35 years of age and treat 90% of those diagnosed. (*3*) However, the ongoing global pandemic of coronavirus disease of 2019 (*4*) has presented challenges to countries in implementing this strategy.

In Viet Nam, cervical cancer is the second most common cancer in women, affecting more than 9000 women from 2016–2017, of whom more than 40% died. (*5*) This is most likely an underestimation due to underreporting of cases in rural Viet Nam. (*1*) In 2016, the Viet Nam Ministry of Health (health ministry) and partners launched the National Action Plan on Prevention and Control of Cervical Cancer 2016–2025, which aims to provide HPV vaccination to 25% of all girls and women, to provide cervical cancer screening to 60% of women aged 30–54 years, to increase early diagnosis of cervical cancer by 40% and to reduce premature cervical cancer mortality by 20% by 2025. (*6*, *7*) These targets have since been deemed unrealistic due to the limited results of cervical cancer prevention and control programmes since the strategic plan was disseminated. (*8*)

HPV is one of the most common sexually transmitted infections (STIs) worldwide, (*9*) with high-risk sexual behaviour being the leading risk factor for infection and subsequent cervical cancer. This includes having multiple sexual partners, early initial sexual intercourse and a compromised immune system. (*10*, *11*) Female sex workers (FSWs) are at high risk of HPV infection due to their having multiple sexual partners. It is also common for them to harbour multiple HPV genotypes and cervical cytological abnormalities. (*12*, *13*) Previous studies in southern and northern Viet Nam found very high HPV prevalence among FSWs (49.5–85%), with the majority (up to 90%) being high-risk HPV types. (*13*, *14*) It was estimated that there are more than 10 000 FSWs in Ho Chi Minh City (HCMC) alone, with the actual numbers to be higher due to challenges in capturing this hard-to-reach population. (*15*) Targeting this high-risk group will be important in reducing the cervical cancer burden in Viet Nam.

The objective of this study was to determine the prevalence of HPV and identify risk factors for high-risk HPV infection among FSWs in Hanoi and HCMC. The findings from this study are expected to inform the Viet Nam health ministry on cervical cancer prevention strategies.

## Methods

### Study design

This cross-sectional study was conducted in collaboration with the HIV/AIDS Centres of Hanoi and HCMC and district health facilities. The study population were women aged 18–50 years old of Vietnamese nationality in Hanoi and HCMC who have been engaging in transactional sex (sex in exchange for money, goods or drugs) in the month before the study. Sample size calculation based on HPV prevalence of 70%, a desired precision of 5%, and a design effect of 2 to address the increase in the variance derived from the cluster design of this survey, determined that 646 FSWs were required to obtain 80% power with a two-sided 5% significance level. A target of 700 (350 FSWs per site) was recruited to allow for 5–10% participant refusal and invalid sample results.

We used a two-stage recruitment strategy. First, four out of 30 administrative districts in Hanoi and five out of 24 administrative districts in HCMC were purposively selected based on the mapping of FSW venues, the FSW population size overseen by the Provincial AIDS Centres, and the participation in HIV sentinel surveillance among FSWs. Within the selected districts, 212 active venues for transactional sex in Hanoi (estimated range of FSWs: 580–1330) and 516 venues in HCMC (estimated range of FSWs: 2700–4800) were identified. Sex work locations included: (i) street-based venues, for example, streets, parks, and other open public places such as under bridges; and (ii) entertainment-based venues, for example, cafes, restaurants, hotels, motels, nightclubs, karaoke lounges, sauna/massage parlours and billiards clubs.

Second, a sampling framework based on the estimated number of FSWs obtained during the mapping exercise was created for the venue-based FSWs. The target subsample sizes for each selected district were proportional to the estimated population size of FSWs, and venues for recruitment were randomly sampled until the sample size was reached. All street-based or entertainment-based FSWs seen at each venue were invited to participate in the study. Visit timing varied across venue types, from daytime for entertainment-based FSWs to night-time for street-based FSWs. Women who were menstruating at enrolment were advised to return and resume their participation after their period had ended. A participant information sheet was provided and written informed consent obtained from all participants.

### Demographic and behavioural data collection

The survey questionnaire included socio-demographic characteristics, smoking, alcohol and/or drug use, sexual behaviours (such as age of sexual debut, sexual acts and sexual partners), menstrual cycle, presence of vaginal bleeding after sex and history of pregnancy. To ensure participants’ confidentiality and safety, face-to-face interviews were conducted in a private room at the district health centres. No identifying information (for example, identity card numbers or addresses) was collected. Late in the study period, sex work-related questions, that is the number of years selling sex and the number of clients in the last month, were added to the survey questionnaire. Each interview lasted approximately 30 minutes. The participants received  80 000 Vietnamese Dongs, approximately US$4, for their participation in this study.

### Clinical examination, specimen collection and HPV screening

A physical and speculum examination was conducted by trained gynaecologists. Cervical swabs were collected and stored in a vial containing 20 ml PreservCyt® Solution (Hologic Inc., MA, United States of America). At the district health centres, specimens collected from Hanoi and HCMC were stored at room temperature and transferred weekly to the National Institute of Hygiene and Epidemiology in Hanoi and the Pasteur Institute in HCMC, respectively. At these institutes, specimens were tested for HPV DNA and sent to the National Hospital of Obstetrics and Gynaecology in Hanoi and the Hung Vuong Hospital in HCMC, respectively, for Papanicolaou (Pap) testing using liquid-based cytology (ThinPrep Pap test, Hologic Inc., ON, Canada). So as to avoid contamination, separate aliquots were used for HPV DNA testing and for cytological examination.

The Bethesda system was used to report Pap smear results, which are categorized as atypical squamous cells of undetermined significance, low-grade squamous intraepithelial lesion, atypical squamous cells, high-grade squamous intraepithelial lesion or squamous cell carcinoma. (*16*) In each city, one cytological technician and one senior cytologist examined the Pap smears with assistance from the ThinPrep imaging system.

### HPV detection and genotyping

HPV detection and genotyping were performed in two steps. First, nucleic acid extraction was performed using the cador Pathogen 96 QIAcube HT Kit (QIAGEN, Hilden, Germany) on an automated platform followed by amplification with PGMY9/11 system by polymerase chain reaction (PCR). (*17*) Positive PCR samples were genotyped using GenoFlow HPV Array test kit (Diagcor Bioscience, Hong Kong Special Administrative Region [China]), which identified 33 HPV types (17 high-risk types: 16, 18, 31, 33, 35, 39, 45, 51, 52, 53, 56, 58, 59, 66/68, 73, 82; 16 low-risk types: 6, 11, 40/61, 42, 43/44, 54/55, 70, 57/71, 72, 81, 84/26). Human leukocyte antigen (HLA) and β-globulin genes were used as internal controls for the PGMY9/11 PCR and Geneflow kit, respectively. Samples negative for the HLA gene were considered invalid and were not included in the analysis. HPV LabNet was used to validate HPV detection and genotyping using 40 study samples from each site: approximately 90% agreement was achieved between the laboratories, as previously reported. (*18*)

### Statistical analysis

We analysed participants’ socio-demographic characteristics and compared FSWs in Hanoi and HCMC using the χ^2^ test or Fisher’s exact test for categorical variables and Student’s *t*-test or the Mann–Whitney U test for continuous variables where appropriate. The prevalence of HPV was unweighted due to the lack of reliable data on size estimates and characteristics of the FSW population in both cities. The exact binomial Clopper-Pearson method was used to estimate 95% confidence intervals (CI) of HPV infection.

HPV types were categorized into high-risk and low-risk, and modified Cox regression analysis was performed to determine factors associated with high-risk HPV infection. The multivariable model included known risk factors for high-risk HPV positivity (for example, smoking), variables with *P* < 0.25 in the bivariate regression models and variables with the Wald statistic of *P* > 0.10 in reduced models. We compared nested models using the likelihood ratio test. We explored co-linearity (for example, between ages at enrolment and sexual debut) and possible interaction terms (for example, between marital status and parturition, and drug use and type of sex worker). Variables with *P*-values £0.05 were considered statistically significant. Data analyses were performed using R software.

## Results

### Participant characteristics

There were 699 FSWs recruited from 67 and 48 active venues for transactional sex in Hanoi and HCMC between December 2017 and May 2018, respectively, with the last 171 participants responding to the additional sex work-related questions.

Participants had a median age of 37 years (range 18–52) and a median age of sexual debut of 19 years (range 11–40). The highest education attained for most participants was secondary school (40.3%). Compared to FSWs in HCMC, a higher number in Hanoi obtained education beyond primary school (*P* < 0.01), had heard of HPV before this study (*P* < 0.01) and lived alone (*P* < 0.01) (**Table 1**). Participants had an average of 11 sexual partners, including both clients and personal partners in the month before the study (**Table 1**).

**Table 1:** Participants’ demographic and behavioural characteristics by city, Hanoi and HCMC, December 2017 to May 2018

Characteristics of female sex workers	Hanoi (*n* = 349)	HCMC (*n* = 350)	Total (*n* = 699)	*P*
**Demographics**	***n*(%)**	***n*(%)**	***n*(%)**	-
Age, in years, median (range)	35 (18–49)	39 (19–52)	37 (18–52)	< 0.001
Age of sexual debut, in years, median (range)	18 (11–27)	19 (14–40)	19 (11–40)	< 0.001
Kinh ethnicity	336 (96.3)	347 (99.1)	683 (97.7)	0.012
Highest education attained				< 0.001
No formal education	5 (1.4)	22 (6.3)	27 (3.9)	-
Primary	51 (14.6)	147 (42)	198 (28.3)	-
Secondary	159 (45.6)	123 (35.1)	282 (40.3)	-
High school or vocational school	115 (33.0)	54 (15.4)	169 (24.2)	-
College or university	19 (5.4)	4 (1.1)	23 (3.3)	-
Marital status				< 0.001
Never married	80 (22.9)	30 (8.6)	110 (15.7)	-
Married	60 (17.2)	106 (30.3)	166 (23.7)	-
Separated	83 (23.8)	45 (12.9)	128 (18.3)	-
Divorced	88 (25.2)	132 (37.7)	220 (31.5)	-
Widowed	38 (10.9)	33 (9.4)	71 (10.2)	-
Living arrangements				< 0.001
With friends	68 (19.5)	39 (11.1)	107 (15.3)	-
With husband, boyfriend, male partner	77 (22.1)	119 (34.0)	196 (28.0)	-
Alone	150 (43.0)	81 (23.1)	231 (33.0)	-
Temporary housing or with family members	54 (15.4)	111 (31.7)	165 (23.6)	-
**Behaviour**				
Ever smoked	72 (20.6)	101 (28.9)	173 (24.7)	0.015
Ever consumed alcohol	230 (65.9)	234 (66.9)	464 (66.4)	0.85
Ever been pregnant	319 (91.4)	306 (87.4)	625 (89.4)	0.068
Number of times pregnant, median (range)	3 (1–20)	2 (1–8)	3 (1–20)	< 0.001
Ever given birth	269 (77.1)	279 (79.7)	548 (78.4)	0.013
Number of times given birth, median (range)	1 (0–5)	1 (0–5)	1 (0–5)	< 0.001
Ever had abortion	253 (72.5)	152 (43.4)	405 (57.9)	< 0.001
Number of abortions, median (range)	2 (1–20)	1 (1–8)	2 (1–20)	< 0.001
Ever used contraception	331 (94.8)	263 (75.1)	594 (85.0)	< 0.001
Consistent condom use	131 (37.4)	114 (32.6)	245 (35.1)	< 0.001
Age started selling sex, median (range)	25 (17–35)	22 (16–42)	24 (16–42)	< 0.001
Number of sexual partners in the last 12 months, median (range)	2 (1–30)	1 (1–65)	1 (1–65)	< 0.001
Number of sex clients in the last month, median (range)^a^	15 (3–31)	8 (0–35)	10 (0–35)	0.006
Street-based sex worker	44 (12.6)	46 (13.1)	90 (12.9)	0.92
Ever consumed drugs	32 (9.2)	34 (9.7)	66 (9.4)	0.9

HCMC: Ho Chi Minh City.

^a^ The number of female sex workers who answered questions related to sex work was 171.

### HPV prevalence and cytology

**Fig. 1:**
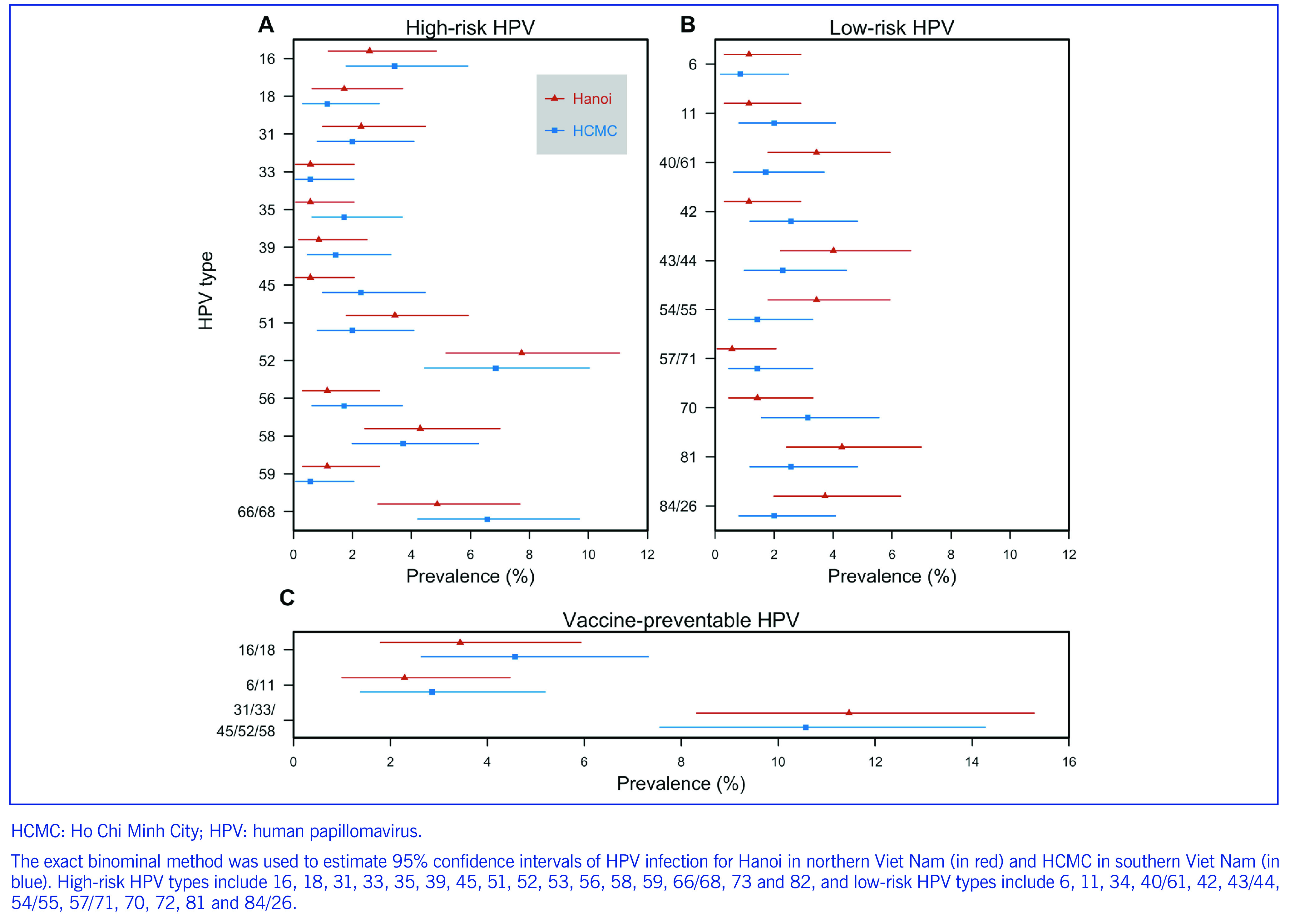
Prevalence of high-risk and low-risk HPV types among female sex workers in Hanoi and HCMC, December 2017 to May 2018

The prevalence of HPV among the 349 FSWs screened in Hanoi was 27.7% and among the 350 FSWs screened in HCMC it was 24.9%. The prevalence of high-risk HPV types was similar between the cities (**Fig. 1**). The prevalence of any high-risk HPV infection was 16.4% (95% CI: 12–21.6%) and 18.2% (95% CI: 14.8–22.1%) for FSWs who reported consistent and inconsistent condom use, respectively. Low-risk HPV types were generally more common among FSWs in Hanoi than HCMC, but this was not statistically significant (**Fig. 1**). HPV type 52 was the most common type (7%) among FSWs in both cities, followed by types 58 (6%) and 66 (4%). The prevalence of infection with multiple HPV types was 18.1% (95% CI: 15.4–21.2%) and was similar between both cities (**Fig. 1**).

[**insert** Figure 1]

The bivariate relationship between Pap cytology and HPV prevalence among the FSWs is shown in  **Table 2**; 13.0% of the FSWs had abnormal Pap cytology and HPV-16/18 accounted for a third of high-grade squamous intraepithelial lesions. In Hanoi, squamous cell carcinoma was identified in a 33-year-old FSW, with HPV-31 present in samples.

**Table 2:** Prevalence of HPV infection by cytological result among female sex workers in Hanoi and HCMC, December 2017 to May 2018

-			Low-grade lesions		High-grade lesions		Cancer
Cytological results	Total *n*(%)	Normal	ASCUS	LSIL	ASC-H	HSIL	SCC
Total^a^	699 (100)	607 (86.8)	52 (7.4)	19 (2.7)	4 (0.6)	15 (2.1)	1 (0.1)
HPV type at time of survey							
16	21 (3.0)	12 (2.0)	1 (1.9)	4 (21.1)	0 (0)	4 (26.7)	0 (0)
18	10 (1.4)	7 (1.2)	1 (1.9)	1 (5.3)	0 (0)	1 (6.7)	0 (0)
16 or 18	28 (4.0)	17 (2.8)	2 (3.8)	4 (21.1)	0 (0)	5 (33.3)	0 (0)
16, 18, 31, 33, 45, 52 or 58	92 (13.2)	65 (10.7)	6 (11.5)	8 (42.1)	2 (50.0)	10 (66.7)	1 (100)
6, 11, 16 or 18^b^	43 (6.2)	29 (4.8)	2 (3.8)	6 (31.6)	0 (0)	6 (40.0)	0 (0)
6, 11, 16, 18, 31, 33, 45, 52 or 58^c^	100 (14.3)	71 (11.7)	6 (11.5)	9 (47.4)	2 (50.0)	11 (73.3)	1 (100)
High-risk HPV types^d^	123 (17.6)	85 (14.0)	9 (17.3)	13 (68.4)	2 (50.0)	13 (86.7)	1 (100)
Low-risk HPV types^e^	109 (15.6)	88 (14.5)	10 (19.2)	8 (42.1)	1 (25.0)	2 (13.3)	0 (0)
Undetermined HPV type	15 (2.1)	12 (2.0)	3 (5.8)	NA	NA	NA	NA

All values are presented as *n*(%). ASC-H: atypical squamous cells cannot exclude HSIL; ASCUS: atypical squamous cells of undetermined significance; HCMC: Ho Chi Minh City; HPV: human papillomavirus; HSIL: high-grade squamous intraepithelial lesion; LSIL: low-grade squamous intraepithelial lesion; NA: not applicable; SCC: squamous cell carcinoma.

The Bethesda system was used to report Pap smear results.

^a^ There was one sample insufficient for cytological testing.

^b^ HPV types covered in the licensed 4-valent vaccine.

^c^ HPV types covered in the licensed 9-valent vaccine.

^d^ High-risk HPV types: 16, 18, 31, 33, 35, 39, 45, 51, 52, 53, 56, 58, 59, 66/68, 73, 82.

^e^ Low-risk HPV types: 6, 11, 34, 40/61, 42, 43/44, 54/55, 57/71, 70, 72, 81, 84/26.

**Fig. 2:**
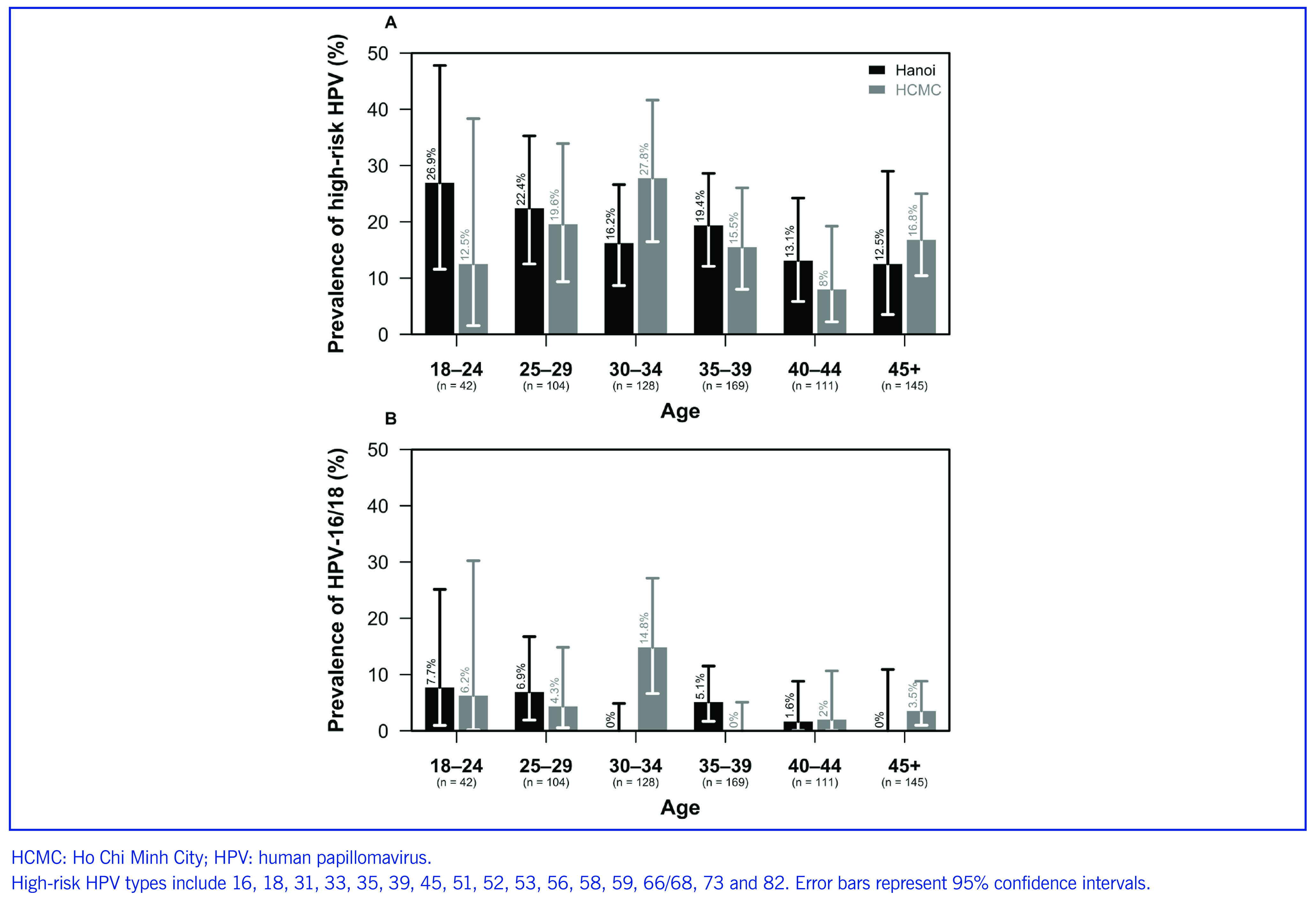
Prevalence of high-risk HPV and HPV-16/18 by age among female sex workers in Hanoi and HCMC, December 2017 to May 2018

High-risk HPV prevalence among younger FSWs (< 25 years old) was higher in Hanoi (27%) than in HCMC (12.5%), while FSWs aged 30–34 years had higher prevalence of high-risk HPV and HPV-16/18 prevalence in HCMC than in Hanoi (**Fig. 2**). Although these analyses were not statistically significant, increasing age was associated with lower risk of both high-risk HPV infection (unadjusted prevalence ratio [PR]: 0.98, 95% CI: 0.96–1, *P* = 0.035) and HPV-16/18 infection (unadjusted PR: 0.95, 95% CI: 0.91–1, *P* = 0.042) (**Table 3**).

[**insert** Figure 2]

**Table 3:** Correlations of factors associated with high-risk HPV infection among female sex workers in Hanoi and HCMC, December 2017 to May 2018

-	Any high-risk HPV^a^				Bivariate analysis		Multivariable analysis	
Variables	N	n	%	*P*	PR (95% CI)	*P*	aPR (95% CI)	*P*
Age	-	-	-	-	0.98 (0.96–1)	0.04	0.98 (0.95–1)	0.06
Age of sexual debut	-	-	-	-	0.94 (0.89–0.99)	0.09	0.95 (0.90–1.01)	0.09
Educational level^b^								
Low	225	44	19.6	0.40	1	-	-	-
High	474	79	16.7	-	0.84 (0.58–1.21)	0.35	-	-
Marital status								
Never married	110	16	14.5	0.44	1	-	1	-
Married	166	32	19.3	-	1.36 (0.75–2.48)	0.31	2.94 (1.29–6.68)	0.01
Separated	128	18	14.1	-	0.96 (0.49–1.89)	0.92	1.48 (0.72–3.05)	0.29
Divorced	220	39	17.7	-	1.24 (0.69–2.22)	0.47	1.96 (1.01–3.81)	< 0.05
Widowed	71	17	23.9	-	1.74 (0.88–3.44)	0.11	3.26 (1.49–7.12)	< 0.01
Other	4	1	25.0	-	1.82 (0.24–13.76)	0.56	3.21 (0.40–25.89)	0.27
Living arrangements								
With friends	107	14	13.1	0.14	1	-	1	-
With husband, boyfriend, male partner	196	33	16.8	-	1.31 (0.70–2.46)	0.39	1.16 (0.54–2.49)	0.70
Alone	231	51	22.1	-	1.78 (0.98–3.21)	0.06	1.85 (1.01–3.39)	< 0.05
Temporary housing or with family members	165	25	15.2	-	1.17 (0.61–2.25)	0.64	1.37 (0.70–2.70)	0.36
Ever smoked	173	32	18.5	0.81	1.08 (0.72–1.61)	0.72	1.23 (0.80–1.88)	0.36
Ever consumed alcohol	464	77	16.6	0.38	0.83 (0.58–1.2)	0.33	-	-
Ever been pregnant	625	102	16.3	0.02	0.54 (0.34–0.87)	< 0.01	-	-
Ever given birth	548	89	16.2	0.09	0.7 (0.47–1.03)	0.07	0.62 (0.38–1.00)	< 0.05
Ever had abortion	405	67	16.5	0.46	0.86 (0.60–1.23)	0.39	-	-
Ever used contraception	594	102	17.1	0.57	0.84 (0.53–1.35)	0.48	-	-
Consistent condom use	244	40	16.4	0.61	0.89 (0.61–1.3)	0.54	-	-
Ever consumed drugs	66	6	9.1	0.08	0.47 (0.21–1.06)	0.07	0.41 (0.17–0.97)	0.04
Type of sex work								
Street-based	90	9	10.0	0.06	1	-	1	-
Venue-based	609	114	18.7	-	1.96 (1–3.87)	0.05	1.85 (0.93–3.67)	0.08
Ever heard of HPV	160	25	15.6	0.54	0.85 (0.55–1.31)	0.46	0.95 (0.90–1.01)	0.09
Ever heard of HPV vaccines	154	29	18.8	0.73	1.1 (0.73–1.67)	0.65	-	-
Ever heard of cervical cancer	325	53	16.3	0.47	0.86 (0.60–1.23)	0.41	-	-
Ever heard of cervical cancer screening	241	36	14.9	0.34	0.72 (0.40–1.28)	0.26	-	-
Study site								
Hanoi, north Viet Nam	349	63	18.1	0.83	1	-	-	-
HCMC, south Viet Nam	350	60	17.1	-	0.94 (0.66–1.35)	0.75	-	-

aPR: adjusted prevalence ratio; HCMC: Ho Chi Minh City; HPV: human papillomavirus; PR: prevalence ratio.

^a^ High-risk HPV types: 16, 18, 31, 33, 35, 39, 45, 51, 52, 56, 58, 59, 66/68.

^b^ Low: primary or no formal education; high: secondary and above.

Approximately 25% of FSWs reported their duration of sex work and number of sex clients in the past month. FSWs in Hanoi who had engaged in sex work for (*3*)20 years had higher prevalence of high-risk HPV than those with < 20 years, whereas FSWs in HCMC who had engaged in sex work for < 10 years had a higher prevalence of high-risk HPV compared to those with (*3*)10 years. Additionally, FSWs who had more clients in the past month had higher prevalence of high-risk HPV (**Supplementary Fig. 1**).

### Risk factors for high-risk HPV infection

FSWs who were married (adjusted PR [aPR]: 2.94, 95% CI: 1.29–6.68, *P* = 0.010), divorced (aPR: 1.96, 95%  CI: 1.01–3.81, *P* = 0.047) or widowed (aPR: 3.26, 95% CI: 1.49–7.12, *P* = 0.003) had higher prevalence of high-risk HPV infection compared to those who were never married. Compared to living with friends, living alone was associated with a higher risk of high-risk HPV infection (aPR: 1.85, 95% CI: 1.01–3.39, *P* = 0.046). In our study cohort, FSWs were less likely to be infected with high-risk HPV if they had given birth (aPR: 0.62, 95% CI: 0.38–1.00, *P* = 0.048) or reported consumption of drugs (aPR: 0.41, 95% CI: 0.17–0.97, *P* = 0.042) (**Table 3**).

The risk factors for FSWs (*n* = 171) who responded to the sex work-related questions are presented in **Supplementary Table 1**; there was no evidence of possible interactions or co-linearity (for example, between age at enrolment and sexual debut) and possible interaction terms (for example, between marital status and parturition, and between drug use and type of sex worker).

## Discussion

In this survey of FSWs in Hanoi and HCMC, the prevalence of HPV was 27.7% and 24.9%, respectively, with almost one in five having high-risk HPV types. HPV types were similar between Hanoi and HCMC with the most common high-risk HPV types being HPV-52, −58 and −66. FSWs who were divorced, widowed or living alone had higher prevalence of high-risk HPV infection. This, as well as being infected with multiple HPV types, has been reported in this group in previous studies, (*19*-*22*) and highlights this group as being susceptible to HPV diseases.

The HPV prevalence observed in our study is lower than previous surveys conducted among FSW populations in Viet Nam (49.5–85%) (*13*, *14*) and other Western Pacific countries (31.6–57.2%). (*23*-*26*) This may be due to several reasons. First, the Global Fund-supported free condom distribution programmes have been largely implemented for FSWs in Viet Nam through community-based organizations, and private and public clinics since 2015. (*27*) Second, our cohort was older and had fewer clients/sexual partners compared to previous studies in southern (*14*) and northern Viet Nam. (*13*) Third, we recruited a higher proportion of venue-based FSWs rather than street-based FSWs, who have higher rates of STIs including HIV. (*28*, *29*) Fourth, the regions are different than in the other studies.

Our finding that one in 10 FSWs had abnormal cytology supports the need for a national cervical cancer screening programme in Viet Nam. WHO recommends screening and treating from age 30, with regular screening every 5–10 years. (*30*) For high-risk women, such as FSWs or those infected with HIV, HPV screening may need to start earlier. (*31*)

HPV-52 was the most prevalent HPV type among FSWs in our cohort, consistent with previous studies of FSWs in Viet Nam. (*13*, *14*) HPV-58 and HPV-66 were the second and third most prevalent types in our cohort. Both HPV-52 and −58 are included in the nonavalent HPV vaccine, while limited cross-protection against these types has been shown from both the bivalent and quadrivalent vaccines. (*32*) This suggests that the nonavalent vaccine may be more appropriate for this high-risk group. HPV vaccination is recommended for individuals before sexual debut, as the vaccines do not clear existing HPV infection, (*33*) although there may still be benefits to women with existing HPV infection, including FSWs. (*34*) These benefits include protection against re-infection and infection with other HPV types, reducing their overall risk of HPV-associated diseases such as cervical cancer, as well as preventing transmission within the community. (*34*)

FSWs who reported consuming drugs or having given birth were less likely to be infected with high-risk HPV; however, these results need to be interpreted with caution. Those who had given birth were almost a decade older than those who had not (median age 38 versus 29 years, respectively), and FSWs who consumed drugs had fewer sexual partners than those who reported no drug use (median 8 versus 10 partners per month, respectively). A review on STIs among FSWs reported an increased risk of infection among drug-using FSWs, possibly due to limited access to health care. (*35*) Furthermore, strict anti-drug laws in Viet Nam discourage disclosing drug consumption among this already vulnerable population, which may have introduced misclassification which could bias the association.

Our study has several limitations. First, we only recruited FSWs from two main cities in Viet Nam, and the prevalence of high-risk HPV was lower than expected. Therefore, caution must be taken in generalizing these findings to the entire population of FSWs in the country. The non-random selection of survey districts in the two cities may have an unweighted procedure for prevalence and characteristics of the FSW population due to the shortage of data on size estimates, leading to additional biases in estimating HPV prevalence among FSWs. Second, our study cohort was older than previous cohorts and most participants were from venue-based work locations, which may not reflect the true HPV prevalence among the FSW population in Viet Nam. However, our cohort may be more representative of persistent HPV infection and risk of cervical cancer. Third, our cohort was selected as they were willing to speak with non-government organizations (NGOs) and public health officials. Since data were incomplete on the response rate among potential participants, the degree to which they were interested in participating in this study is unknown. Hence, findings from this study should be interpreted with caution. Fourth, self-reporting during face-to-face interviews could limit the reliability of information on sexual risk behaviours and drug use. Fifth, our study did not record the vaccination status of FSWs. Some may have been vaccinated because of other research studies or small pilot vaccination programmes, thus directly or indirectly protecting them from HPV-16 and −18 infection. Lastly, the small number of FSWs with high-grade squamous intraepithelial lesions or cervical cancer limited our ability to identify their association with HPV infection.

## Conclusions

We found high prevalence of high-risk HPV infection among FSWs in Hanoi and HCMC, highlighting the need for a targeted HPV prevention campaign. We recommend HPV prevention strategies such as screening every 5–10 years from age 25 as previously described by WHO30 and HPV vaccination targeting this vulnerable group of women. These strategies will protect FSWs from HPV-associated diseases including cervical cancer, and also help to reduce HPV transmission within the community and the overall cervical cancer burden in Viet Nam and other LMICs with similar settings.
